# Acute Effects of Breath-Hold Conditions on Aerobic Fitness in Elite Rugby Players

**DOI:** 10.3390/life14080917

**Published:** 2024-07-23

**Authors:** Wendi Wang, Dongzhe Wu, Hao Wang, Zhiqiang Zhang, Xuming Jiang, Shufeng Li, Yongjin Shi, Xiaolin Gao

**Affiliations:** 1 Sports Rehabilitation Research Center, China Institute of Sport Science, Beijing 100061, China; wangwendi@ciss.cn (W.W.); wdz276640188@outlook.com (D.W.); wanghao@ciss.cn (H.W.); 2 School of Sport Science, Beijing Sport University, Beijing 100084, China; 3 Department of Sports and Arts, China Agricultural University, Beijing 100083, China; johnny1974@sina.cn (Z.Z.); jxm7762@126.com (X.J.); lishufeng@cau.edu.cn (S.L.); shiyongjin@cau.edu.cn (Y.S.)

**Keywords:** breath-hold, apnea, diving reflex, warm-up, aerobic fitness, cardiopulmonary optimal point, autonomic nervous system, peak oxygen uptake, stroke volume

## Abstract

The effects of face immersion and concurrent exercise on the diving reflex evoked by breath-hold (BH) differ, yet little is known about the combined effects of different BH conditions on aerobic fitness in elite athletes. This study aimed to assess the acute effects of various BH conditions on 18 male elite rugby players (age: 23.5 ± 1.8 years; height: 183.3 ± 3.4 cm; body mass: 84.8 ± 8.5 kg) and identify the BH condition eliciting the greatest aerobic fitness activation. Participants underwent five warm-up conditions: baseline regular breathing, dynamic dry BH (DD), static dry BH (SD), wet dynamic BH (WD), and wet static BH (WS). Significant differences (*p* < 0.05) were found in red blood cells (RBCs), red blood cell volume (RGB), and hematocrit (HCT) pre- and post-warm-up. Peak oxygen uptake (VO_2_peak) and relative oxygen uptake (VO_2_/kgpeak) varied significantly across conditions, with BH groups showing notably higher values than the regular breathing group (*p* < 0.05). Interaction effects of facial immersion and movement conditions were significant for VO_2_peak, VO_2_/kgpeak, and the cardiopulmonary optimal point (*p* < 0.05). Specifically, VO_2_peak and peak stroke volume (SVpeak) were significantly higher in the DD group compared to that in other conditions. Increases in VO_2_peak were strongly correlated with changes in RBCs and HCT induced by DD warm-up (r∆RBC = 0.84, r∆HCT = 0.77, *p* < 0.01). In conclusion, DD BH warm-up appears to optimize subsequent aerobic performance in elite athletes.

## 1. Introduction

Breath-hold (BH) is frequently utilized in sports such as diving and swimming to improve athletes’ endurance. This practice has been associated with notable erythropoietic responses, leading to increased hemoglobin concentration and potentially enhancing athletic performance through the diving reflex (DR) [1,2]. DR involves bradycardia, decreased cardiac output, increased vascular resistance, and the redistribution of blood flow [3,4]. Its primary function is to reduce blood flow and oxygen supply to nonessential organs, redirecting resources to oxygen-dependent organs. Recent research has demonstrated that even a single maximal breath-hold can enhance athletic performance [5,6,7]. However, understanding the diverse factors influencing BH and their impact on athletic performance remains an ongoing area of investigation [8,9].

Initially, the DR was thought to be triggered exclusively by BH with facial immersion, stimulating the trigeminal nerve [10]. Emerging research indicates that a similar reflex can be elicited by BH even without facial immersion, leading to related physiological effects. Consequently, research on BH’s physiological effects has predominantly focused on specific factors such as facial immersion [11], water temperature [12], and dive depth [13]. Nonetheless, caution is warranted regarding the potential adverse effects of cold-water-induced trigeminal nerve over-stimulation, which may excessively activate the parasympathetic nervous system and impede subsequent exercise performance.

Additionally, static BH differs from dynamic BH in several important ways. Static BH induces bradycardia and ensures blood supply to vital organs through α-adrenergic vasoconstriction [14], which constricts blood vessels, reducing oxygen consumption and cardiac load. In contrast, dynamic BH is followed by a higher heart rate (HR) [15], increased oxygen consumption, and blood redistribution to cardiac and skeletal muscles [16]. Moreover, BH, while reflexively enhancing muscle tone, is believed to augment muscle strength [17], facilitating more efficient activation of muscle groups and improving the body’s energy expenditure rate. Thus, dynamic BH may produce superior physiological effects on DR [18]. However, current studies only compare the effects of regular lower limb exercise and face immersion individually, neglecting their combined impact or interactions. Given BH’s significant impact on physiological parameters, it is crucial to investigate how these changes translate into athletic performance, especially during high-intensity exercise. The athletes’ exercise performance serves as a critical dependent variable in understanding the practical applications of BH strategies in sports.

This study hypothesized that combining facial immersion (wet and dry) and movement conditions (dynamic and static) during breath-holding affects cardiopulmonary fitness in elite athletes. The primary objective was to explore how these different conditions influence athletes’ exercise performance during subsequent high-intensity exercise. To achieve this, we selected cardiopulmonary exercise tests, hemodynamic parameters, and hematological parameters as key measures, providing a comprehensive evaluation of the physiological responses and their potential implications for performance. Additionally, the study aimed to assess the feasibility of incorporating BH into the warm-up process and investigate related physiological mechanisms.

## 2. Materials and Methods

### 2.1. Procedures

Sample size calculation was conducted using G*Power 3.1 software, confirming the sufficiency of the sample size for this study. Statistical power analysis indicated that, with a significance level (α) of 0.05, a medium effect size (Cohen’s d = 0.65), and a statistical power of ≥0.7, a total of 17 participants were required for the survey.

The study utilized a self-controlled trial design, wherein each participant completed all five warm-up protocols in a randomized order across five separate trials, with a one-week interval between each trial. Participants’ random trial sequence was determined by labeling the experiments 1–5 and using Microsoft Excel 2010. Before each trial, all subjects performed a uniform lower limb muscle stretch for 3 min and then entered a 10 min warm-up intervention phase. Fingerstick blood was collected before and after each warm-up intervention. Cardiopulmonary exercise testing was started immediately after the warm-up intervention. To account for possible circadian rhythm effects, all subjects were tested between 9:30 and 11:00 a.m. to ensure that both experiments on the same subject were conducted during the same time period. Participants were instructed not to consume caffeine or alcoholic beverages within the 48 h preceding the test day.

### 2.2. Participants

Eighteen male athletes (age: 23.5 ± 1.8 years; height = 183.3 ± 3.4 cm; BM: 84.8 ± 8.5 kg; BMI: 25.2 ± 2.0 kg/m^2^) were voluntarily recruited to participate in this study. Prior to the commencement of the formal trial, participants were briefed on the trial’s complete process and objectives. They provided informed consent by signing a consent form and underwent exercise risk screening. Additionally, participants were instructed to abstain from consuming caffeine or alcohol within the 48 h preceding the test day. The day before each experimental condition, participants were ensured a minimum of eight hours of sleep and were required to wear identical sneakers and clothing during the experiment. An experimental technician supervised the entirety of the exercise test. The Ethics Committee of the China Institute of Sports Science approved the experiment.

The inclusion criteria were as follows: ① being aged 18–25 years old, holding a national second-level or above athlete qualification certificate; ② being healthy and without recent movement disorders; ③ having no cardiovascular or cerebrovascular disease, and no family history of sudden death; ④ understanding the trial, signing the informed consent form, and committing to participate in and complete the entire trial process.

The exclusion criteria were as follows: ① a presence of cardiac, cerebral, or vascular diseases and a family history of sudden death; ② failure of routine ECG, blood pressure monitoring, and blood screening; ③ underlying medical problems or a history of ankle, knee, or back injuries; ④ any lower extremity reconstructive surgery or unresolved musculoskeletal disorders in the last two years; ⑤ previous BH dives or hypoxic training; ⑥ travel to a high-altitude area in the last six months.

### 2.3. Testing Procedures

When the subjects arrived at the laboratory, they initially sat quietly for 3 min. Following this period, they performed a 3 min lower limb muscle stretch designed to reduce injury risk during subsequent cycling. This was followed by pedaling on the power bike, during which various BH procedures were employed to assess the impact of different environmental factors on exercise performance.

**Figure 1:**
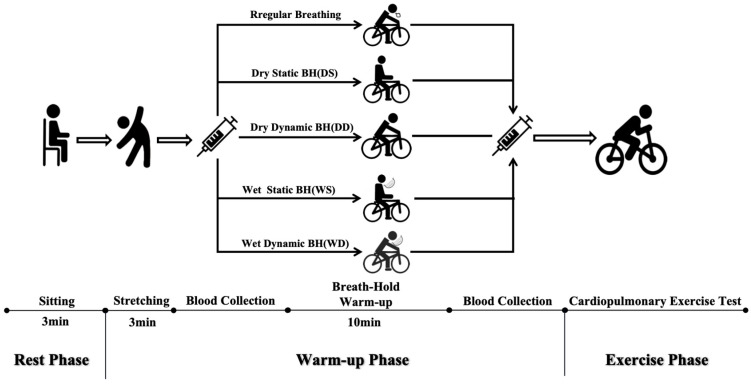
Procedures.

The specific phases of the cardiopulmonary exercise test were as follows: (1) The quiet phase: 2 min of sitting still on the bike. (2) The first blood collection phase: peripheral blood collection from fingertips. (3) The warm-up phase: five different warm-up interventions were performed. (4) The second blood collection phase: peripheral blood collection from fingertips. (5) The exercise phase: the initial load of 40 W was maintained for 2 min, followed by a linear load increment of 25 W/min. The electronic tachometer controlled the pedaling speed at 60–70 rpm, with verbal encouragement provided. Participants meeting any one of these criteria were regarded as having reached the maximum load [19,20], which entailed the following: ① reaching 90% of the individual’s maximum predicted HR (calculated as 210 − (age × 0.65)) or maintaining a stable HR for two minutes; ② reaching a rate of perceived exertion (RPE) ≥ 17; ③ showing a decline in oxygen uptake during [21] sustained exercise changes or a decrease in oxygen uptake during continuous exercise. (6) The recovery stage: pedaling at 20 W for 3 min followed by equipment unloading. (7) The end stage: subjects were allowed to leave the laboratory only after a 30 min rest with no observed abnormalities. After the 30 min rest period, subjects were permitted to leave the laboratory if no abnormalities were detected. The experimental flow is shown in Figure 1.

### 2.4. Testing Parameters

Cardiorespiratory exercise tests were conducted using a vertical power bike (Eegoselect100, Ergoline Academy, Würzburg, Germany) equipped with a telemetric exercise cardiorespiratory tester (Cortex MetaMax3B, Leipzig, Germany). The system measured gas exchange parameters including peak oxygen uptake (VO_2_peak), peak oxygen uptake per kilogram of body weight (VO_2_/kg peak), the anaerobic threshold (AT), and the anaerobic threshold per kilogram of body weight (AT/kg) [19]. Ventilatory efficiency, indicated by the lowest ventilatory equivalent ratio for oxygen (VE/VO_2_), highlights cardiac or pulmonary limitations during exercise and formed a “U” curve during an incremental load exercise test. The lowest point, known as the cardiopulmonary optimal point (COP) [22], serves as a submaximal indicator to assess the cardiorespiratory efficiency of athletes [23,24].

We calculated deltas (Δ) to observe the differences between regular warm-up and breath-hold warm-up conditions. Each Δ was determined by subtracting the value measured under regular warm-up conditions from the value measured after the breath-hold warm-up intervention. Specifically, the calculations were as follows:

(1) ΔVO_2_peak = VO_2_peak (BH warm-up) − VO_2_peak (Regular warm-up);
(2) ΔVO_2_/kg peak = VO_2_/kg peak (BH warm-up) − VO_2_/kg peak (Regular warm-up);
(3) ΔCOP = COP (BH warm-up) − COP (Regular warm-up).

Additionally, a noninvasive cardiac output system (Cheetah NICOM, USA) was used to measure cardiovascular indicators at VO_2_peak, including peak stroke volume (SVpeak), peak heart rate (HRpeak), and peak cardiac output (COpeak). Similarly, we calculated the deltas for additional key variables to assess differences in SVpeak, HRpeak, and COPEAK. These calculations were as follows:

(1) ΔSVpeak = SVpeak (BH warm-up) − SVpeak (Regular warm-up);
(2) ΔHRpeak = HRpeak (BH warm-up) − HRpeak (Regular warm-up);
(3) ΔCOpeak = COpeak (BH warm-up) − COpeak (Regular warm-up).

Hematological parameters were collected by professionals following the Chinese Consensus on the Operation of Capillary Blood Collection. Fingerstick blood samples were immediately transferred to the laboratory to measure various blood parameters, including red blood cells (RBCs), hemoglobin (HGB), and hematocrit (HCT). We used Δ to represent the changes in RBCs, HGB, and HCT before and after the warm-up intervention. Each Δ was calculated by subtracting the value measured before the warm-up from the value measured after the warm-up. Specifically, the calculations were as follows:

(1) ΔRBC = RBC (post-warm-up) − RBC (pre-warm-up);
(2) ΔHGB = HGB (post-warm-up) − HGB (pre-warm-up);
(3) ΔHCT = HCT (post-warm-up) − HCT (pre-warm-up).

### 2.5. Warm-Up Methods

Five warm-up methods were used for eighteen male athletes, one of which was a regular breathing warm-up, and the remaining four of which were BH warm-ups. The baseline regular warm-up was 10 min of zero-power pedaling while breathing normally, with all pedaling maintained at 60–70 r/min. The dynamic dry BH (DD) group performed a warm-up of 10 min of zero-power pedaling at the same time lasting for six times the maximum BH; static dry BH (SD) required the subject to sit quietly on a power bike for 10 min while performing the procedure for a duration of six- times the maximum BH; wet dynamic BH (WD) entailed 10 min of zero-power pedaling at the same time for a duration of six times the maximum BH when the participant’s face was immersed in water; wet static BH (WS) required the subject to be on a power bike for 10 min while submerging their face in water for a duration of six times that of the BH.

All BH warm-ups involved six repetitions of maximal voluntary end-expiratory breath-holds, with a 30 s interval between each BH. BH commenced at the end of natural expiration, with participants instructed not to initiate the hold immediately after maximal inhalation. The criterion for each BH pause was that the impulse to resume breathing was more significant than the willingness to hold the breath before the subject could resume free breathing. Wet BH (including WD and WS) alternated between face immersion in cold water for maximal BH and free breathing with the face held above the water surface, with only the neck flexed during the face immersion BH to ensure that the entire face was immersed in water. Dry BH (DD & SD) refers to the maximal BH in the air with the neck flexed to hold the body in almost the same position. The water temperature was maintained between 10 and 11 °C.

### 2.6. Safety Monitoring

Throughout the entire testing procedure, heart rate was continuously monitored using a Polar heart rate monitor (Polar Electro, Kempele, Finland). Testing was discontinued if any three of the following conditions were met [19]: (1) the increment in oxygen uptake between two consecutive sessions was less than 2 mL/(kg·min) or it decreased; (2) the respiratory quotient was equal to or greater than 1.15; (3) the HR exceeded 180 beats per minute or did not increase within 2 min. During breath-hold phases, participants were closely observed for signs of hypoperfusion, such as changes in lip or fingertip color, cold sweats, or dizziness.

### 2.7. Statistical Analyses

Data were processed using SPSS 26.0 (IBM, Chicago, IL, USA) software. GraphPad Prism 9.0 was used to construct figures. Normality was examined with the Shapiro–Wilk test. Homoscedasticity was determined through Levene’s test. Analysis was performed using a two-way repeated measures ANOVA [25] in the facial immersion condition (dry and wet) × 2-movement condition (dynamic and static) in four BH warm-up groups for gas metabolism parameters and hemodynamic parameters. When significant main or interaction effects were observed, pairwise comparisons were performed using the Bonferroni post hoc test. Effect size and partial eta squared (η2) are presented where appropriate. Paired sample *t*-tests were conducted between the regular group and each of the four BH warm-up groups. A paired sample *t*-test was performed on RBCs, HCT, and HGB before and after five warm-ups to explore the hematological changes. Pearson correlation analyses were performed to determine the relationship between ΔVO_2_peak and ΔRBC, ΔHCT, and ΔHGB. A general linear regression model was established when r > 0.5. The significance level was set at *p* < 0.05. *p* = 0.000 was reported as *p* < 0.001. Data are reported as means ± SD.

## 3. Results

All athletes completed the protocol without adverse reactions. All data passed the Shapiro–Wilk test and Levene’s test. 

### 3.1. Cardiopulmonary Exercise Test Parameters

**Figure 2:**
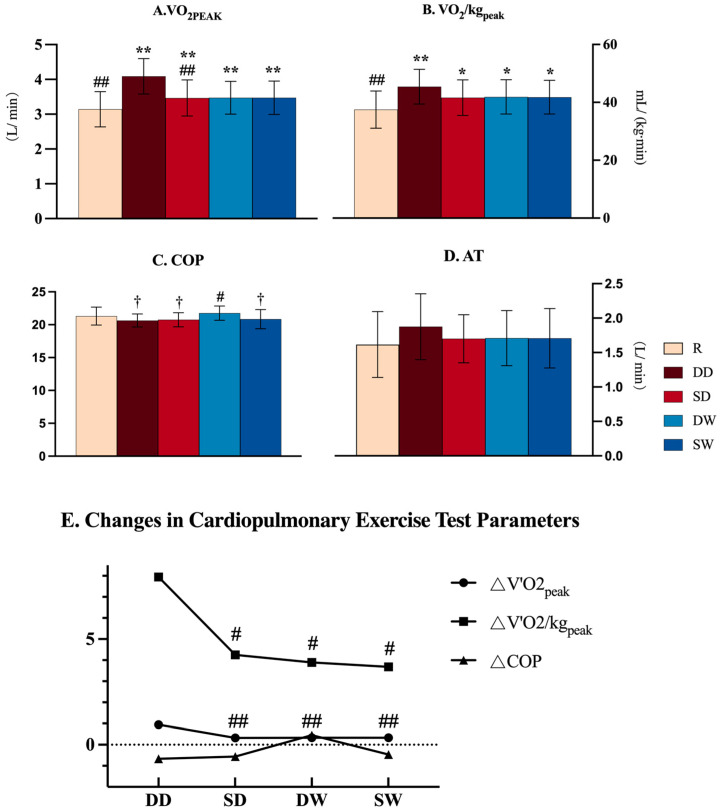
Gas metabolism parameters. R = regular breathing warm-up; DD = dynamic dry breath-hold; SD = static dry breath-hold; WD = dynamic wet breath-hold; WS = static wet breath-hold; VO_2_peak = peak oxygen uptake; VO_2_/kgpeak = relative peak oxygen uptake; COP = cardiopulmonary optimal point; AT = anaerobic threshold; △VO_2_peak, △VO_2_/kg peak, and △COP = BH warm-up(VO_2_peak, VO_2_/kg peak, COP)-Regular (VO_2_peak, VO_2_/kg peak, COP); significant difference vs. regular breathing group (* *p* < 0.05, ** *p* < 0.01); significant difference vs. DD group (# *p* < 0.05, ## *p* < 0.01); significant difference vs. DW group ( † *p* < 0.05).

Two-way repeated measures ANOVA revealed significant between-group differences in VO_2_peak and VO_2_/kgpeak (*p* < 0.001, Figure 2A,B). Static conditions exhibited a significantly lower VO_2_peak values compared to Dynamic conditions (F = 52.4, *p* < 0.001, Static < Dynamic, Figure 2A). VO_2_/kgpeak in all four BH groups was significantly higher than in the regular breathing group (*p* < 0.05, Figure 2B). COP after the WD warm-up was significantly higher than in the other BH groups (*p* < 0.05, Figure 2C). There was no significant difference in AT among the five groups (all *p* > 0.05, Figure 2D). Dry conditions exhibited significantly higher ΔVO_2_peak values compared to wet conditions (F = 39.9, *p* < 0.001, Dry > Wet, Figure 2E). There was no significant intergroup difference in ΔCOP (*p* = 0.178, Figure 2E).

There was a significant effect of the facial immersion condition on VO_2_peak, VO_2_/kgpeak, and COP (*p* < 0.05). There was also a significant effect of the movement condition on VO_2_peak and VO_2_/kgpeak (*p* < 0.05). A significant interaction between the facial immersion condition and movement condition was found for VO_2_peak, VO_2_/kgpeak, and COP (*p* < 0.05, Table 1).

### 3.2. Hemodynamic Parameters

**Figure 3:**
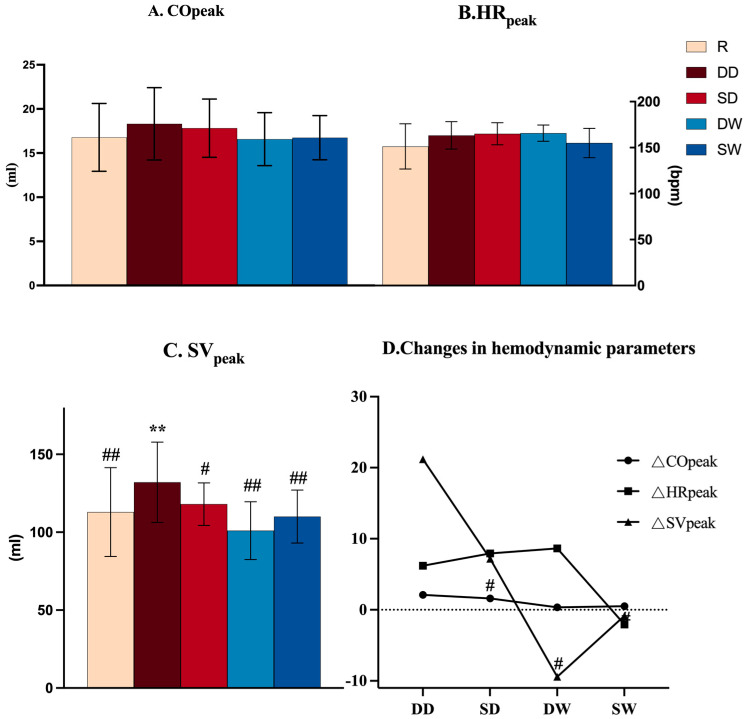
Hemodynamic parameters. R = regular breathing warm-up; DD = dynamic dry breath-hold; SD = static dry breath-hold; WD = dynamic wet breath-hold; WS = static wet breath-hold; COpeak = peak cardiac output; HRpeak = peak heart rate; SVpeak = peak stroke volume. ΔSVpeak = SVpeak (BH warm-up)−SVpeak (regular warm-up); ΔHRpeak = HRpeak (BH warm-up)−HRpeak (regular warm-up); ΔCOPEAK = COPEAK (BH warm-up)−COPEAK (regular warm-up). Significant difference vs. regular breathing group (** *p* < 0.01); significant difference vs. DD group (# *p* < 0.05, ## *p* < 0.01).

Analysis of variance with the groups as factors showed no significant differences in COpeak and HRpeak among the five groups (*p* > 0.05, Figure 3A,B). The SVpeak of the DD group was significantly higher than that at the baseline and that of the other three BH warm-up groups (Figure 3C). The DD group showed higher ΔSVpeak values compared to the other three BH warm-up groups (*p* < 0.05, Figure 3D).

Statistical results revealed a significant interaction (Facial immersion × Movement) upon reaching SVpeak and HRpeak (*p* < 0.05). Dynamic HRpeak was higher than static HRpeak under wet conditions (*p* = 0.025).There was significant effect of the facial immersion condition on SVpeak and COpeak (*p* < 0.05).There was no significant effect of the movement condition on all hemodynamic parameters (*p* > 0.05) (Table 1).

### 3.3. Hematological Parameters

**Figure 4:**
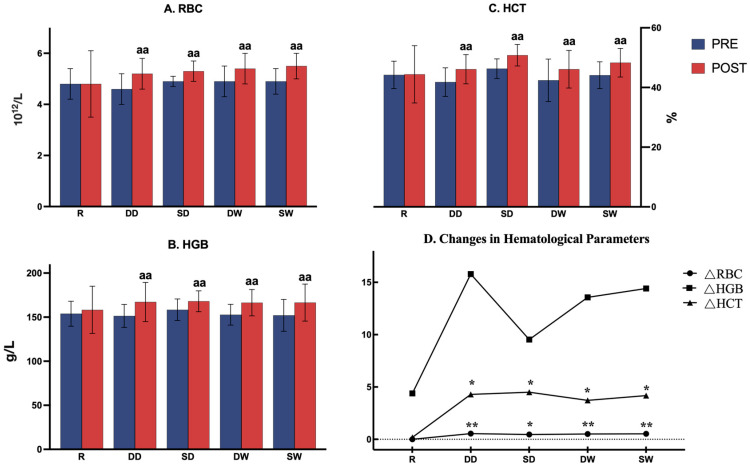
Hematological parameters. R = regular breathing warm-up; DD = dynamic dry breath-hold; SD = static dry breath-hold; WD = dynamic wet breath-hold; WS = static wet breath-hold; RBC = red blood cells; HGB = hemoglobin; HCT = hematocrit. ΔRBC = RBC (post-warm-up)−RBC; ΔHGB = HGB (post-warm-up)−HGB (pre-warm-up); ΔHCT = HCT (post-warm-up)−HCT (pre-warm-up). Significant difference vs. pre-breath-hold warm-up (aa *p* < 0.01); significant difference vs. regular breathing group (* *p* < 0.05, ** *p* < 0.01).

Before warm-up initiation, there were no significant differences observed among the five groups for RBCs, HGB, and HCT (*p* > 0.05). Paired-sample *t*-tests on RBCs, RGB, and HCT before and after the warm-up in four BH groups showed significant differences (*p* < 0.05, Figure 4A–C). ΔRBC and ΔHCT were significantly higher after the BH warm-up interventions compared to the corresponding indicators after the regular warm-up (*p* = 0.021, Figure 4D).

### 3.4. VO_2_peak Correlation and Regression Analysis

**Figure 5:**
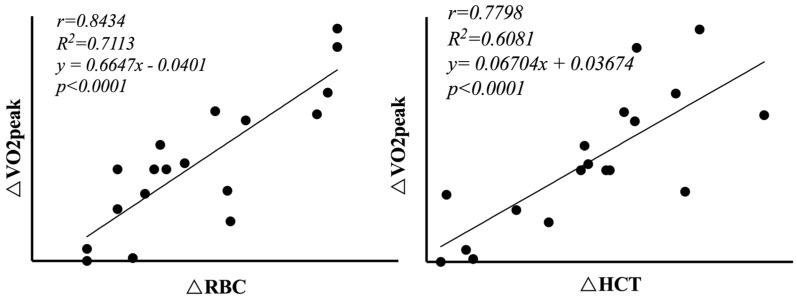
Linear regression model.

There was a significant correlation between △VO_2_peak and △RBC, △HCT in the DD group (rRBC = 0.84, rHCT = 0.77, *p* < 0.01). There was no significant correlation between △HGB and △VO_2_peak.The linear regression model is detailed in Figure 5.

## 4. Discussion

To our knowledge, this is the first study to investigate the effects of four BH conditions on subsequent cardiopulmonary fitness in elite athletes. Our findings showed the the following: (1) there was a significant interaction between facial immersion conditions and movement conditions during BH warm-ups; (2) compared with other BH warm-ups, the dynamic dry (DD) warm-up significantly enhanced oxygen uptake and transportation, increased circulatory efficiency, and improved cardiovascular function, showing the most pronounced effect on cardiopulmonary fitness improvement. Therefore, these results strongly suggest that the DD warm-up can be used as an effective warm-up modality, offering better body activation effects.

### 4.1. Cardiorespiratory

The study findings indicate that VO_2_/kgpeak following the BH warm-up surpassed that of the regular warm-up group. Previous research suggests that BH impacts various cardiorespiratory parameters, including cardiac output, venous oxygen reserve, and pulmonary oxygen reserve. Traditionally associated with decreased cardiac output and oxygen reserves, recent studies indicate potential variations in these effects [1,26]. Pre-exercise oxygen storage levels play a significant role in determining cardiopulmonary fitness. The development of warm-up methods targeting enhanced oxygen storage before exercise underscores the importance of optimizing this parameter. These findings underscore the potential for alternative warm-up strategies to improve VO_2_peak through enhanced oxygen availability.

The study highlights the influence of environmental factors, such as movement conditions and facial immersion, on VO_2_/kg peak. Specifically, it demonstrates that the movement conditions and facial immersion condition collectively explain 72.2% of the variability. Notably, dry BH (4.1 L/min) resulted in higher VO_2_peak compared to wet BH(3.5 L/min) under similar movement conditions, suggesting a potential interaction between facial immersion and warm-up methods. Previous research suggests that the relationship between sympathovagal balance and cardiopulmonary fitness significantly influences athletic performance [27]. Pre-exercise parasympathetic dominance positively affects cardiorespiratory parameters and endurance exercise performance, such as running [28]. However, an imbalance between sympathetic and parasympathetic inputs to cardiac autonomic regulation may delay the withdrawal of parasympathetic nerves before exercise, subsequently affecting the timely activation of sympathetic nerves and reducing cardiorespiratory fitness [29]. This aligns with our study’s outcomes, suggesting that DD BH may have a more robust interaction effect, thereby pre-activating sympathetic activity and favoring pre-exercise autonomic activation preparation.

Research indicates that higher CO_2_ tolerance correlates with a lower COP, suggesting enhanced cardiorespiratory endurance [24]. The present study showed an interaction effect between facial immersion conditions and movement conditions for COP [30]. Under movement conditions, COP reduction after a dry BH was significantly higher than that after wet BH (*p* = 0.001). During incremental exercise, VO_2_ increases linearly to a plateau, whereas ventilation shows a sharp inflection point of increase [22]. Higher PaCO2 stimulates peripheral chemoreceptors, inducing reflexive deep and fast respiration and increased blood circulation [31]. Dry BH reduces COP by increasing PaCO_2_ earlier, increasing CO_2_ tolerance, leading to a non-equivalent proportional change in VE and VO_2_. Dry BH as a warm-up method may reduce COP and enhance respiratory oxygen consumption during exercise, improving overall cardiorespiratory efficiency.

A meta-analysis has shown that BH training facilitates improved anaerobic glycolysis [32]. Mechanisms may include increases in glycogen and phosphocreatine availability, enzyme activity, buffering capacity, or improved tolerance to exercise-induced acidosis [33]. Training adaptations to anaerobic glycolysis may include an increase in the maximal rate of lactate production and an improvement in muscle buffering capacity, leading to an increase in lactate clearance and minimizing pH disturbances in muscle [34]. The lack of direct measurement of blood lactate concentrations in this paper may have limited the exploration of improvements in anaerobic capacity, suggesting that future studies consider adding pre- and post-warm-up blood lactate measurements. Additionally, rugby players were selected as subjects for this study. The typical training emphasis of rugby players on high-intensity explosive activities such as sprinting, jumping, and tackling may shape their partial anaerobic threshold predominantly through their training regimen, potentially resulting in minimal impacts from a single BH warm-up. This could explain why the study did not detect significant differences following various warm-up modalities.

### 4.2. Hemodynamic Parameters

Different warm-up types induce changes in hemodynamic parameters upon reaching VO_2_peak, primarily affecting stroke volume (SV) and HR. Following DD warm-up, SVpeak demonstrates a notable increase compared to that in other warm-up methods. Engaging in a DD BH warm-up before exercise enhances SV, thereby improving oxygen delivery capacity. Helgerud et al. suggested that enhanced cardiac contractility contributing to increased SV may explain the observed enhancement in cardiopulmonary fitness [35]. Increased SVpeak indicates improved blood pumping efficiency, crucial for rugby players requiring adequate oxygen and nutrient supply to muscles during intense exercise, thus sustaining athletic performance. Additionally, Woorons et al. noted that maximal dry BH may prolong heart filling time, augment left ventricular filling, and reduce right ventricular volume, thereby increasing left ventricular output and improving oxygen delivery to muscles [36].

Furthermore, this study’s results revealed that facial immersion conditions significantly influenced SVpeak and COpeak, explaining 51.4% and 22% of their variability, respectively (*p* < 0.05). Submerging the face in water during dynamic warm-up decreased SVpeak, possibly due to increased parasympathetic activation from the DR, counteracting sympathetic activation during high-intensity exercise. Specifically, the DD group showed a significantly higher increase in SVpeak (ΔSVpeak) compared to the other three BH groups and the conventional warm-up group. Higher SVpeak indicates enhanced oxygen and nutrient delivery to muscles, critical for maintaining performance during intense competition and training.

There was a significant interaction effect (facial immersion × movement) on HRpeak (*p* < 0.05). BH and facial immersion increase parasympathetic tone, decreasing HR, while exercise onset stimulates sympathetic activation, leading to increased HR [29]. Typically, HR increases approximately 5–10 s after exercise onset due to the delay between the stimulus and physiological response [37,38]. In contrast, dynamic BH initiates HR changes at the BH onset (i.e., exercise start) due to DR activation.

Under wet conditions, the dynamic HRpeak was higher than the static HRpeak (*p* = 0.025). Butler and Woakes reported no significant difference between static and dynamic BH under dry conditions [39]. Sterba et al. conducted a study using 20 °C water cooling during static BH, resulting in a 12% HR decrease during and a 19% decrease after dynamic BH [40]. However, unlike previous studies focusing on recovery post-BH, ours assessed HRpeak during exercise immediately following BH. During the transition from warm-up to exercise, HR initially decreases due to reduced parasympathetic activation, followed by sympathetic tone increase. Previous research focused on factors affecting BH like water temperature, body area submerged, cooling method (immersion or spray) [41], BH mode (end-expiration or end-inspiration) [42], diving experience [43], and work-related activities, among others. Thus, future research can study the BH impact on cardiopulmonary fitness, controlling HR via the autonomic nervous system using indicators like HR variability.

High-lung-volume BH leads to a sudden intrapulmonary pressure rise, decreasing cardiac preload and increasing afterload, temporarily lowering CO, and reducing blood and oxygen supply to tissues. Continued ventilation restores sympathetic nerve dominance, increasing HR and contractility, restoring or slightly increasing CO to normal levels. COpeak defines cardiorespiratory fitness. Repeated end-expiratory BH warm-ups slightly increased COpeak compared to conventional warm-ups, consistent with prior research [20].

### 4.3. Hematologic Parameters

Previous research has demonstrated that prolonged breath-hold (BH) training induces physiological adaptations, including increased splanchnic volume, erythrocyte pressure volume, erythropoietin (EPO) production, hemoglobin (HGB) levels, and lung capacity [44,45,46]. These adaptations have been associated with improvements in aerobic fitness and maximal exercise capacity [47]. In our study, significant changes in red blood cell (RBC), HGB, and hematocrit (HCT) levels were observed before and after BH, indicating an acute response to hypoxic stimulation. This suggests that BH may enhance erythrocyte reserve and improve oxygen transport capacity, potentially contributing to improved aerobic fitness.

The DR induced by BH results in splenic contraction, causing a transient surge in hemoglobin concentration and erythrocyte pressure volume [48]. This response enhances oxygen carrying capacity and redirects blood flow from peripheral vasculature to critical organs such as the brain and heart. The Valsalva effect during BH may further trigger peripheral vasoconstriction, facilitating blood redistribution and optimizing oxygen delivery [49]. However, these effects are transient, typically lasting for 10 min after BH cessation. Multiple BH sessions, as employed in our study, may accumulate these effects, potentially leading to a more pronounced oxygen conservation effect post-warmup. Schagatay et al. concluded that this effect persists for at least 10 min after BH, suggesting that maximal exercise following BH warm-up can effectively utilize enhanced erythrocyte reserve and exhibit higher aerobic fitness [50].

Furthermore, the Valsalva effect induced during BH may trigger peripheral vasoconstriction, directing blood from the peripheral vasculature towards the central circulatory system, thereby increasing the concentration of RBCs within a given blood volume. Dynamic BH redistributes blood while inducing selective sympathetic-induced vasoconstriction in small arteries, particularly in peripheral and visceral capillary beds of nonessential organs and body extremities. This results in the preferentially oxygenated blood in critical organs such as the brain and heart. Consequently, nonessential organs shift metabolically from predominantly aerobic to predominantly anaerobic metabolism, further enhancing the oxygen conservation effect mediated by the diving response. However, due to a circulatory delay in oxygen storage within peripheral veins, this effect typically diminishes within 15 to 45 s following BH cessation. In our study, participants performed six maximal BH exercises at 30 s intervals, potentially accumulating delayed oxygen storage effects and leading to a more pronounced oxygen conservation effect post-warmup.

Another important finding of this study is that different conditions impact BH-induced splanchnic contraction, affecting erythrocyte circulation. Elia et al. demonstrated that dynamic dry BH induces more pronounced splanchnic contraction. In their study of eight non-apneic divers, they found no significant change in EPO concentration 30 min after the BH intervention compared to the baseline (6.62 ± 3.03 mlU/mL vs. 8.46 ± 2.21 mlU/mL; *p* = 0.109) [51]. Espersen et al. found an immediate decrease in the relative content of erythrocytes following static wet BH [52]. Conversely, Bouten et al. found that static dry BH led to a more significant elevation in HGB compared to dynamic dry BH. Furthermore, some studies have not observed changes in erythrocyte circulation-related parameters following a single BH [53].

In our study, SD BH elicited an increase in RBC from 4.9 to 5.3 × 10^12^/L, while DD BH led to an increase from 4.6 to 5.2 × 10^12^/L. Although both conditions resulted in higher RBC levels compared to the baseline, the difference was not statistically significant. During static BH without limb movement, core muscles contract more, increasing intra-abdominal pressure and exerting more pressure on the spleen. Consequently, this leads to a greater release of HGB and RBCs into the circulatory system. However, excessive erythrocyte release prior to exercise may elevate erythrocyte pressure production, increase blood viscosity, and slow blood flow, all detrimental to oxygen carrying capacity.

Notably, Bouten et al. concluded that acute BH did not improve performance in professional cycling over a 3 km distance. Similarly, Sperlich et al. supported that four BH sessions preceding a 4 km cycling time trial resulted in splanchnic constriction without notable changes in hemoglobin levels, erythrocyte pressure production, lateral femoral muscle oxygen saturation, or oxygen uptake [40]. In contrast, the robust correlation observed between changes in ∆RBC and ∆HCT were induced by dynamic BH warm-up and alterations in ∆VO_2_/kg peak in our study. Research indicates that both animal and recent human studies are consistent in demonstrating a spleen contraction of 40–70% during maximal exercise. Regardless of whether dry or wet breath-holding is employed, the spleen contraction induced exceeds 25%. Qvist et al. found hematological changes occurring only with significant spleen contraction, defined as more than 25%, whether conducted underwater or in a laboratory setting with BH [54]. This study, limited by the mode of exercise, did not immediately measure spleen volume after BH. However, given the significant relationship between ∆RBC and ∆HCT under ∆VO_2_/kg peak, we can reasonably infer that BH warm-up induces changes in erythrocyte circulation by eliciting spleen contraction.

### 4.4. Application in Training

Dynamic dry BH warm-up sessions should be strategically integrated into athletes’ training routines, particularly before high-intensity workouts or competitions. Our study demonstrates that dynamic dry BH significantly enhances oxygen uptake, improves circulatory efficiency, and optimizes cardiovascular function, thereby enhancing immediate cardiopulmonary readiness. Coaches and trainers can tailor the duration and intensity of these sessions based on individual athlete fitness levels and specific sport demands, ensuring gradual adaptation and maximizing physiological benefits over time.

It is crucial to provide athletes with educational support regarding the physiological mechanisms underlying dynamic dry BH. This understanding not only enhances their compliance with the warm-up protocol but also fosters appreciation of its impact on performance. Regular monitoring of athletes’ responses, including metrics such as heart rate variability, recovery rates, and perceived exertion, allows for adjustments to optimize warm-up effectiveness while minimizing potential risks associated with BH practices, such as the overstimulation of the parasympathetic nervous system [55].

Moreover, emphasizing safety protocols, especially concerning cold water exposure during warm-up procedures, ensures that the implementation of dynamic dry apnea warm-ups remains safe and effective. By following these guidelines, coaches and athletes can leverage the benefits identified in our study to optimize athletic performance through a scientifically supported warm-up strategy that enhances cardiopulmonary fitness and overall readiness.

### 4.5. Limitations

Our study focused on elite rugby players, known for their confrontational sport demands and distinct physiological characteristics, such as intermittent high-intensity activities, frequent collisions, and diverse movement patterns. These factors can significantly influence how athletes respond to warm-up strategies like dynamic dry BH, impacting its effectiveness in enhancing cardiopulmonary fitness outcomes. While our findings offer valuable insights into this specific athlete group, their direct applicability to other athletes, particularly those with different physiological adaptations and training focuses, may be limited. To enhance the generalizability of our results, future studies should expand participant inclusion across various sports and disciplines. This approach would provide a broader understanding of how dynamic dry BH warm-up strategies affect different physiological profiles and athletic demands.

## 5. Conclusions

BH warm-up serves as an effective preparatory measure, significantly enhancing oxygen uptake and delivery, improving circulatory efficiency, and enhancing cardiovascular function. Variations in BH conditions, such as the presence or absence of facial immersion in cold water and dynamic activity, result in different physiological effects. Among these conditions, dynamic dry BH shows the most promising potential for improving immediate cardiopulmonary fitness. Our findings underscore the value of dynamic dry BH warm-up as a beneficial preparatory strategy, positively impacting athletes’ cardiopulmonary fitness. Future research should explore the detailed physiological mechanisms and optimal protocols of dynamic dry BH warm-up, comparing its effectiveness with traditional warm-up methods. Additionally, assessing its long-term impact on fitness, performance, and psychological factors will provide a comprehensive understanding of its benefits for athletes.

## Figures and Tables

**Table 1:** Warm-up condition effect. VO_2_peak = peak oxygen uptake; VO_2_/kgpeak = relative peak oxygen uptake; COP = cardiopulmonary optimal point; AT = anaerobic threshold; AT/kg = relative anaerobic threshold; COpeak = peak cardiac output; HRpeak = peak heart rate; SVpeak = peak stroke volume. “×” represents the interaction effects of the two conditions on the parameters.

	Facial Immersion Condition			Movement Condition			Facial Immersion Condition × Movement Condition		
	F(1,17)	Partial η^2^	*p*	F(1,17)	Partial η^2^	*p*	F(1,17)	Partial η^2^	*p*
VO2peak (L/min)	39.942	0.755	<0.001	52.405	0.701	<0.001	95.239	0.849	<0.001
VO2/kg peak (mL/(kg·min)	13.011	0.434	0.002	17.984	0.514	0.001	44.197	0.722	<0.001
COP	5.765	0.253	0.028	2.501	0.128	0.132	5.223	0.235	0.035
AT (L/min)	1.695	0.091	0.21	1.102	0.061	0.308	1.064	0.059	0.317
AT/kg (mL/(kg·min))	5.401	0.241	0.033	0.889	0.05	0.359	1.637	0.088	0.218
SVpeak	17.98	0.514	0.001	0.75	0.042	0.398	7.176	0.297	0.016
HRpeak	1.911	0.101	0.185	1.986	0.105	0.177	9.157	0.35	0.008
COpeak	4.804	0.22	0.043	0.119	0.007	0.119	0.206	0.012	0.656

## Data Availability

The data used in this study are available from the corresponding author upon reasonable request.
